# Broad impact of extracellular DNA on biofilm formation by clinically isolated Methicillin-resistant and -sensitive strains of *Staphylococcus aureus*

**DOI:** 10.1038/s41598-018-20485-z

**Published:** 2018-02-02

**Authors:** Shinya Sugimoto, Fumiya Sato, Reina Miyakawa, Akio Chiba, Shoichi Onodera, Seiji Hori, Yoshimitsu Mizunoe

**Affiliations:** 1 Department of Bacteriology, The Jikei University School of Medicine 3-25-8, Nishi-Shimbashi, Minato-ku, Tokyo, Japan; 2 Jikei Center for Biofilm Research and Technology, The Jikei University School of Medicine 3-25-8, Nishi-Shimbashi, Minato-ku, Tokyo, Japan; 3 Department of Infectious Disease and Control, The Jikei University School of Medicine 3-25-8, Nishi-Shimbashi, Minato-ku, Tokyo, Japan

## Abstract

*Staphylococcus aureus* is a major causative agent for biofilm-associated infections. Inside biofilms, *S*. *aureus* cells are embedded in an extracellular matrix (ECM) composed of polysaccharide-intercellular adhesins (PIA), proteins, and/or extracellular DNA (eDNA). However, the importance of each component and the relationship among them in biofilms of diverse strains are largely unclear. Here, we characterised biofilms formed by 47 *S*. *aureus* clinical isolates. In most (42/47) of the strains, biofilm formation was augmented by glucose supplementation. Sodium chloride (NaCl)-triggered biofilm formation was more prevalent in methicillin-sensitive *S*. *aureus* (15/24) than in methicillin-resistant strain (1/23). DNase I most effectively inhibited and disrupted massive biofilms, and Proteinase K was also effective. Anti-biofilm effects of Dispersin B, which cleaves PIA, were restricted to PIA-dependent biofilms formed by specific strains and showed significant negative correlations with those of Proteinase K, suggesting independent roles of PIA and proteins in each biofilm. ECM profiling demonstrated that eDNA was present in all strains, although its level differed among strains and culture conditions. These results indicate that eDNA is the most common component in *S*. *aureus* biofilms, whereas PIA is important for a small number of isolates. Therefore, eDNA can be a primary target for developing eradication strategies against *S*. *aureus* biofilms.

## Introduction

Microbes commonly form a well-organised and complex community on surfaces, known as biofilms, to survive in diverse environments^1,2^. Biofilm formation proceeds from initial contact of an individual microorganism with a surface. Later, the cells attached to the surface proliferate and form a micro-colony on such surfaces. An extracellular matrix (ECM) composed of proteins, polysaccharides, and/or extracellular DNA (eDNA) is produced, and a complex, three-dimensional structure is established to form a mature biofilm^3^. Maturation stage is followed by the release of microbial cells within the biofilm through dispersal, which results in reactivation of a planktonic state^4,5^. Inside a biofilm, microbial cells are embedded in ECM, which binds them to each other and to the substrate. ECM maintains structural integrity of biofilms and protects microbes from environmental stresses, attacks by other organisms (*e*.*g*., host immune system), and by chemical agents (*e*.*g*., antibiotics)^2^. Antibiotic tolerance in biofilms is also associated with the emergence of isogenic subpopulations of antibiotic-sensitive bacteria that acquire tolerance to multiple antibiotics due to stochastically induced dormant states^6,7^. Therefore, when biofilms are formed on human organs or on medical devices, such as central venous catheters and pacemakers, they frequently cause chronic infectious diseases^8^.

*Staphylococcus aureus* is a commensal bacterium that colonises mammalian skin or mucous membranes, and is also an opportunistic pathogen, causing hospital-acquired, surgical-site, and medical device-associated infections^9^. Previously, it was reported that environmental conditions, including composition of culture media, affected biofilm-forming capacity and matrix components in staphylococcal biofilms^10,11^. For example, brain-heart infusion (BHI) medium, one of the nutrient-rich laboratory media, and tryptic soy broth (TSB) medium, a less nutrient-rich medium, which are frequently used for biofilm research, were shown to affect matrix composition in staphylococcal biofilms. The quantity of proteins in the matrix was greater in BHI medium than in TSB, whereas TSB induced production of polysaccharides, termed as PIA or poly N-acetylglucosamine (PNAG)^10^. Glucose and NaCl were shown to promote biofilm formation by various *S*. *aureus* strains via acidification of culture media (due to secretion of acidic metabolites)^12^ and via induction of PIA production^11^, respectively. Moreover, relationship between antibiotic resistance and biofilm phenotypes is also of interest, since both contribute to the success of staphylococci as human pathogens in clinical conditions. O’Gara’s group reported that methicillin-sensitive *S*. *aureus* (MSSA) commonly produces a biofilm that depends on PIA^11^. In contrast, PIA-independent biofilms were more pronounced in methicillin-resistant *S*. *aureus* (MRSA) than those in MSSA^11^. However, it is still unclear whether this is the case in other strains isolated from different places (*e*.*g*., hospitals, cities, and countries).

Importance of eDNA in biofilms has been proposed in diverse bacterial species, including *S*. *aureus*, based on susceptibility of biofilms to DNase I. It was reported that DNase I inhibited biofilm formation of a PIA-independent MRSA clinical isolate, but did not inhibit a PIA-dependent MSSA strain^13^. In that study, DNase I was effective to inhibit the formation of the MRSA biofilm but not to disperse the pre-formed biofilm^13^. On the other hand, another study demonstrated that DNase I could prevent biofilm formation by a PIA-dependent MSSA as well as PIA-independent MRSA and could disperse the preformed biofilms^14^. However, these studies were conducted only in limited number of strains, and the association between characteristics of eDNA, PIA-dependency in biofilm formation, and methicillin-resistance are not fully understood. To address this, roles of eDNA in biofilm development and structural integrity need to be comprehensively evaluated in diverse strains. In addition, clarification about the amount and properties of eDNA in the biofilms is also important.

In the present study, we examined the effects of media composition on biofilm-forming capacities of *S*. *aureus* strains isolated in Jikei University Hospital and investigated potential associations among biofilm phenotypes, methicillin resistance, and genetic backgrounds. We also analysed susceptibility of biofilms to ECM-degrading enzymes and levels of eDNA in biofilms. We found that eDNA has broad impacts on the development and structural integrity of both PIA-dependent and -independent biofilms. Importantly, the amount of eDNA was not always correlated with biofilm-forming capacity, suggesting that small but significant amount of eDNA is sufficient for development of robust biofilms. Taken together, these results provide significant insights into mechanisms of biofilm formation and roles of eDNA in *S*. *aureus*, which may contribute to the development of strategies to eradicate *S*. *aureus* biofilms.

## Results

### Glucose and NaCl trigger biofilm formation by clinically isolated strains of *S*. *aureus*

We investigated the effects of media compositions on biofilm formation by *S*. *aureus*, a major causative bacterium of biofilm-associated infections in clinical conditions. Initially, biofilm formation of *S*. *aureus* RN4220 wild-type and its isogenic ∆*icaA* mutant strain were analysed and designated as positive and negative controls, respectively. These strains were cultivated in BHI or TSB media with or without supplementation with glucose or NaCl (referred as BHIG, BHIN, TSBG, and TSBN, respectively). The biofilms, which were formed on the flat-bottom surfaces of 96-well microplates, were stained with crystal violet (CV) according to conventional procedures^15^. Biofilm phenotypes of these strains were distinct depending upon the composition of culture media (Fig. S1). The wild-type strain formed massive biofilms in BHIN, TSBG, and TSBN media, whereas the ∆*icaA* strain formed biofilms only in TSBG medium. These results indicate that RN4220 forms PIA-dependent biofilms in NaCl-supplemented media, whereas it produces PIA-independent biofilms in glucose-supplemented media. Therefore, cultivation in different media was useful to understand whether *S*. *aureus* produces a PIA-dependent or PIA-independent biofilm.

**Figure 1:**
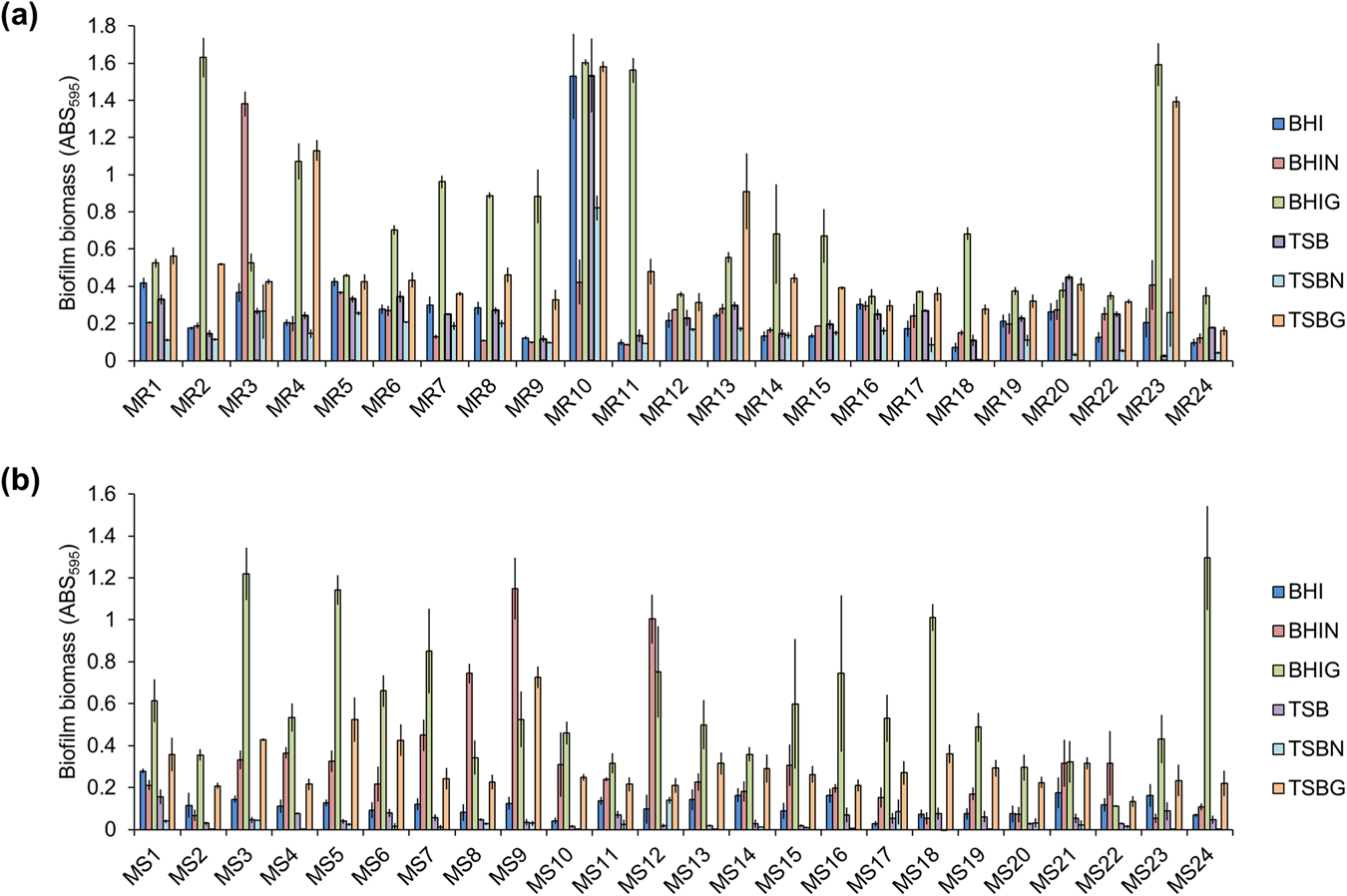
Biofilm biomass of various clinically isolated *S*. *aureus* strains under different culture conditions. Biofilms of the indicated strains of (**a**) MRSA and (**b**) MSSA were formed in the indicated media at 37 °C for 24 h. Y-axes represent biofilm biomass determined by measuring absorbance of CV-stained biofilms at 595 nm. Data are represented as mean ± standard deviation from at least three independent experiments.

Next, 23 MRSA and 24 MSSA strains, isolated from various sites of patients in Jikei University Hospital (Table S1), were cultivated in BHI, BHIG, BHIN, TSB, TSBG, or TSBN media. As illustrated in Fig. 1, biofilm biomass differed among strains. There was no significant difference in biofilm-producing capacity between MRSA and MSSA grown in media with glucose (*P* = 0.249) or NaCl (*P* = 0.591) (Fig. S2). We also determined the genetic background of *S*. *aureus* strains, in terms of clinical significance, by accessory gene regulator (*agr*) typing and multi-locus sequence typing (MLST) (Table S2). However, there was no statistically significant relationship between biofilm-forming capacity and *agr* types (*P* = 0.265) (Fig. S2). On the other hand, more than half of MLST types are represented by only one strain. Thus, no conclusions about the association between biofilm formation and MLST type can be reached.

Notably, almost all strains (97.9%, 46/47) formed more elaborate biofilms in the BHI-based media than in the TSB-based media, except for MR13 strain whose biofilm biomass was the highest in TSBG medium. Glucose remarkably promoted biofilm formation in diverse strains, as biofilms formed by 89.4% of the isolates (20/23 MRSA and 22/24 MSSA) were stimulated by glucose (Fig. 1). On the other hand, effect of NaCl was comparatively limited, as NaCl promoted biofilm formation by 62.5% of MSSA strains (15/24) but only by 4.3% of MRSA strains (1/23). This indicated that the NaCl-triggered biofilm formation was more prevalent among MSSA than in MRSA isolates (*P* < 0.0001). Interestingly, only MR10 strain formed a robust biofilm in both BHI as well as TSB media without supplementation of glucose or NaCl, while its biofilm development was in fact partially inhibited by NaCl (Fig. 1).

Based on the highest value of the biofilm biomass depicted in Fig. 1, the strains were categorised into high (ABS_595_ ≥ 0.8), medium (0.4 < ABS_595_ < 0.8), or low (ABS_595_ ≤ 0.4) biofilm producers. Accordingly, 39.1% of MRSA (9/23) and 20.8% of MSSA (5/24) isolates were categorised as high in the presence of glucose; 4.3% of MRSA (1/23) and 8.3% of MSSA (2/24) isolates were as high in the presence of NaCl; 34.8% of MRSA (8/23) and 50% of MSSA (12/24) isolates were as medium in the presence of glucose; 13.0% of MRSA (3/23) and 8.3% of MSSA (2/24) isolates were as medium in the presence of NaCl; 26.1% of MRSA (6/23) and 29.2% of MSSA (7/24) isolates were as low in the presence of glucose; 82.6% of MRSA (19/23) and 83.3% of MSSA (20/24) isolates were as low biofilm producers in the presence of NaCl (Table S2).

### Susceptibility of biofilms formed by *S*. *aureus* clinical isolates to ECM-degrading enzymes

Contribution of ECM components to biofilm robustness can be readily examined by evaluation of susceptibility of biofilms to ECM-degrading enzymes. Here, we used Proteinase K, DNase I, and Dispersin B for degradation of major constituents, such as proteins, eDNA, and PIA, respectively. To gain clearer insights into the effects of these enzymes, we selected the high biofilm producers that include 10 MRSA and 7 MSSA strains. For biofilm inhibition assay, enzymes were added to culture media at the onset of biofilm formation, whereas to perform destruction assay, enzymes were added to mature biofilms at 24 h. The bacteria were cultured in media supplemented with glucose or NaCl.

**Figure 2:**
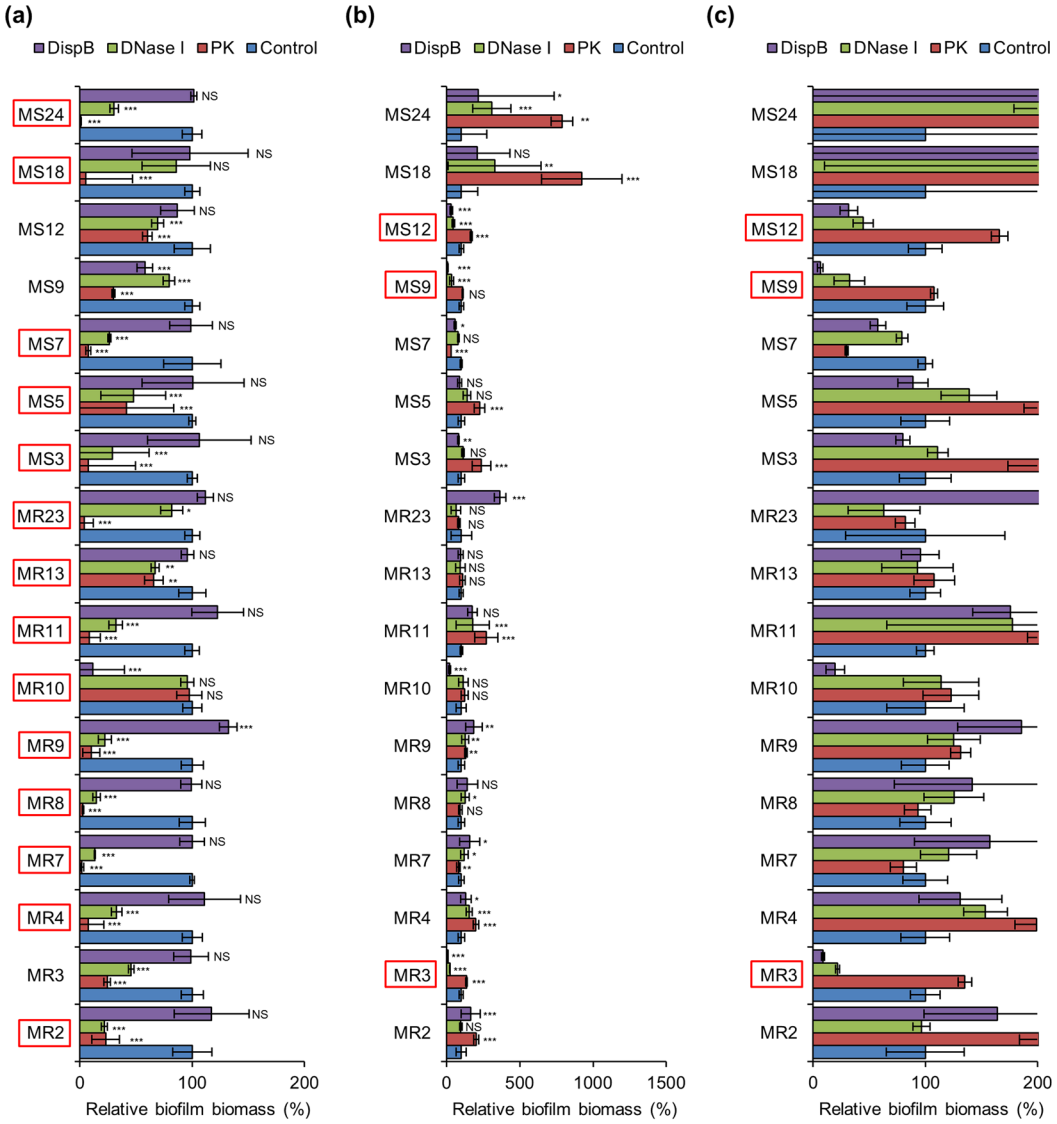
Inhibition of biofilm formation by ECM-degrading enzymes. Biofilms of the high-biofilm producing *S*. *aureus* strains were formed in BHIG (except for MR13 that was cultured in TSBG) (**a**) and BHIN (**b**) supplemented with Proteinase K, DNase I, and Dispersin B or non-supplemented (control). Biofilm biomass was quantified after 24 h. (**c**) For easier evaluation, the range of the X-axis of **b** was adjusted. All strains marked with red squares were cultured in the optimal medium for biofilm formation (Fig. 1). X-axes represent the percentage of biofilm biomass determined by measuring absorbance of CV-stained biofilms at 595 nm. Controls were defined as 100%. Data are represented as mean ± standard deviation from at least three independent experiments. *P < 0.05; **P < 0.01; ***P < 0.001; NS, not significant.

**Figure 3:**
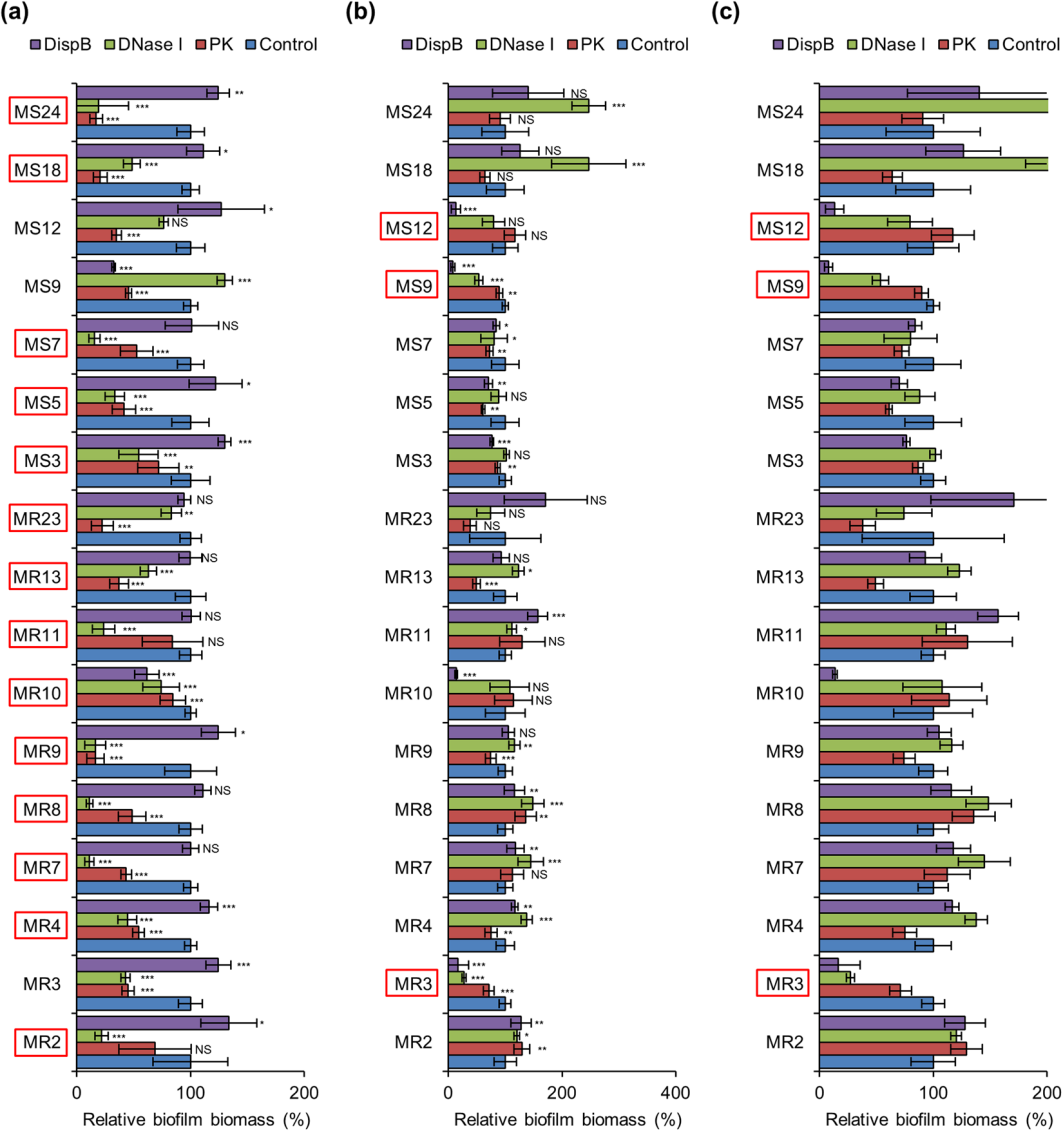
Dispersal of biofilms by ECM-degrading enzymes. Biofilms formed for 24 h in BHIG (except for MR13 that was cultured in TSBG) (**a**) and BHIN (**b**) were treated with or without the indicated enzymes at 37 °C for 2 h. (**c**) For easier evaluation, the range of the X-axis of **b** was adjusted. The residual biofilms were quantified. All strains marked with red squares were cultured in the optimal medium for biofilm formation (Fig. 1). X-axes represent the percentage of biofilm biomass determined by measuring absorbance of CV-stained biofilms at 595 nm. Controls were defined as 100%. Data are represented as mean ± standard deviation from at least three independent experiments. *P < 0.05; **P < 0.01; ***P < 0.001; NS, not significant.

When cultured in glucose-supplemented media, almost all biofilms were inhibited (16/17) and dispersed (15/17) by Proteinase K (Figs 2a and 3a). Similar proportion of biofilms were prevented (15/17) and destructed (15/17) by DNase I (Figs 2a and 3a). Under the same condition, Dispersin B showed limited anti-biofilm activities against certain strains (2/17 for inhibition and 2/17 for dispersal) (Figs 2a and 3a). On the other hand, in NaCl-supplemented media, a small number of biofilms were prevented (6/17) and destructed (7/17) by Dispersin B (Figs 2b and 3b). Unexpectedly, Proteinase K was also effective for inhibiting (1/17) and dispersing (8/17) biofilms cultivated in NaCl-supplemented media (Figs 2b and 3b). In addition, DNase I prevented (3/6) and degraded (3/7) PIA-dependent biofilms formed in the presence of NaCl (Figs 2b and 3b). However, it should be noted that these comparisons were conducted with data obtained from the results including very small amounts of biofilms formed under non-optimal conditions (Fig. 1). To combat biofilm-associated issues, control of robust and massive biofilms must be important. Therefore, we focused on the effects of the enzymes on large amounts of biofilms (ABS_595_ ≥ 0.8) formed in the optimal medium for each strain (Fig. 1) as described below.

DNase I was the most effective enzyme to antagonise diverse massive biofilms, as it significantly prevented biofilm generation in 88.2% (15/17, except for MR10 and MS18) of the strains tested (Fig. 2) and dispersed 94.1% (16/17, except for MS12) of the preformed biofilms (Fig. 3). These results suggest that eDNA is the most common and important component in massive *S*. *aureus* biofilms and that it is not only essential for the initial attachment of bacteria but also for the maturation and robustness of biofilms. Proteinase K also showed effective anti-biofilm activities. It inhibited biofilm formation in 76.5% (13/17, except for PIA-dependent biofilms of MR3, MR10, MS9, and MS12) of the isolates (Fig. 2) and destroyed 82.4% (14/17, except for MR2, MR11, and MS12) of the preformed biofilms (Fig. 3). In contrast to the other enzymes, Dispersin B showed limited anti-biofilm activities. It prevented biofilm generation and destructed the preformed biofilms in 23.5% (4/17) of the strains (Figs 2 and 3), suggesting that PIA plays a key role only in limited strains (MR3, MR10, MS9, and MS12).

Among the tested strains, MR2, MR10, MR11 and MR23 showed a specific enzyme-susceptibility. As previously reported, the MR23 biofilm was sensitive to Proteinase K^15–17^ but highly resistant to DNase I and Dispersin B (Figs 2a and 3a), whereas the MR10-derived biofilm was remarkably inhibited and slightly dispersed by Dispersin B (Figs 2 and 3). On the other hand, MR2 and MR11 biofilms preformed in BHIG were resistant to both Proteinase K and Dispersin B and sensitive to DNase I (Fig. 3a). These results indicate that PIA and proteins are the primary component in the biofilms of MR10 and MR23, respectively, while eDNA is crucial for the MR2 and MR11 biofilms grown with glucose.

**Figure 4:**
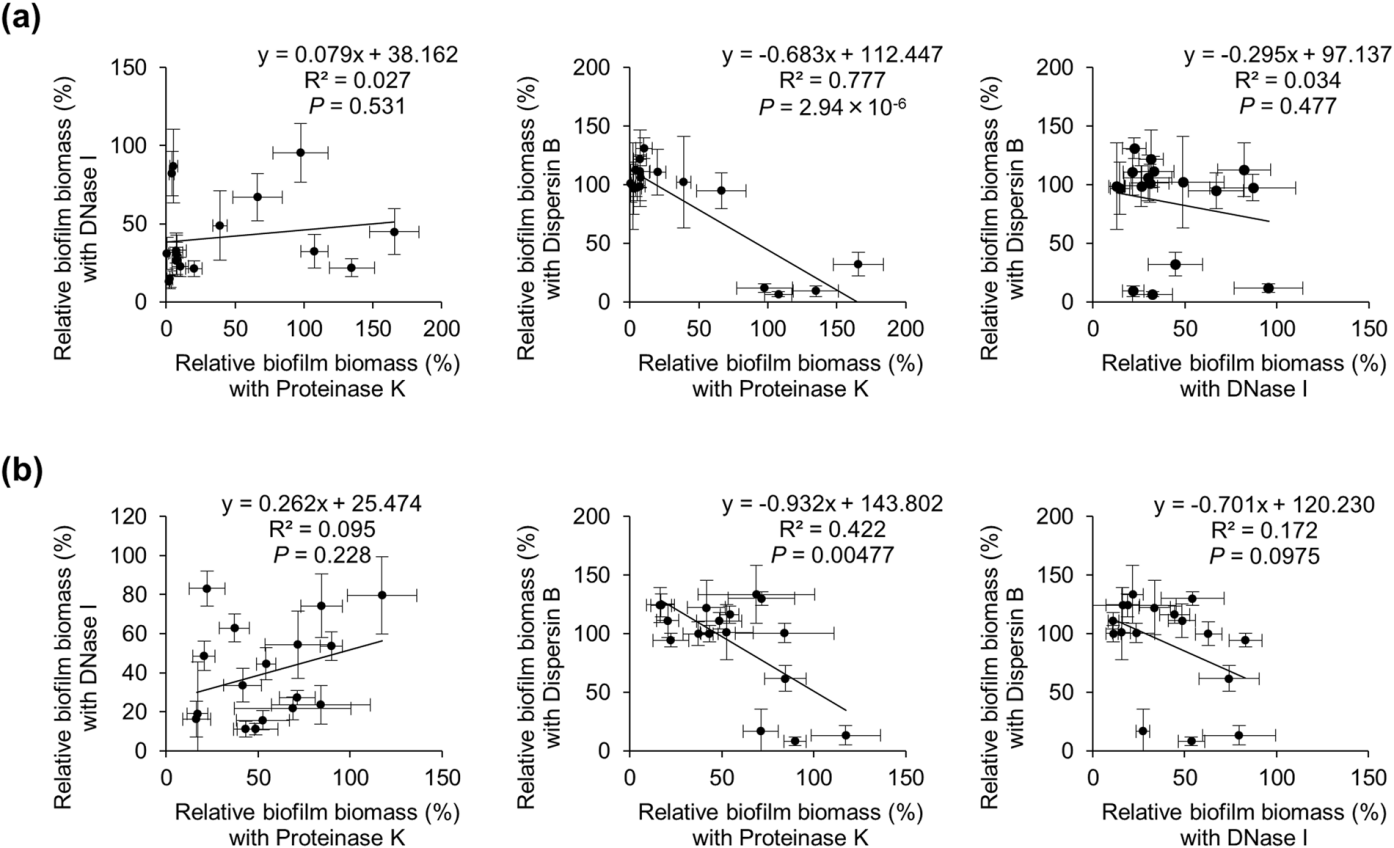
Correlation of anti-biofilm activities among enzymes. (**a**) The means and standard deviations from biofilm inhibition assay (Fig. 2) were plotted using the data under the optimal biofilm conditions. Correlations of biofilm biomasses formed in the presence of the indicated enzymes are shown. (**b**) The means and standard deviations from biofilm destruction assay (Fig. 3) were plotted using the data under the optimal biofilm conditions. Correlations of biofilm biomasses after treatment with the indicated enzymes are shown. The correlation coefficient (R^2^) was determined by fitting the data as a linear model using Microsoft Excel 2010.

Interestingly, biofilm inhibition activities of Proteinase K and Dispersin B revealed a significant negative correlation (R^2^ = 0.777, middle panel of Fig. 4a), suggesting that proteins and PIA play independent roles in massive biofilms. DNase I showed no significant correlation with the other enzymes (left and right panels of Fig. 4a) due to broad-spectrum inhibitory activity of DNase I. The results of biofilm destruction assay also exhibited similar trends (Fig. 4b). These results suggest that treatment with multiple ECM-degrading enzymes, including DNase I, would be a promising approach to inhibit or eradicate extensive biofilms.

### eDNA profiles in *S*. *aureus* biofilms

ECM components in bacteria are thought to play key roles in biofilm development and structural integrity. Therefore, characterisation of ECM is important for understanding the molecular mechanisms underlying biofilm establishment. According to the results of enzyme-susceptibility assay, eDNA likely played a key role in the development and structural integrity of biofilms formed by diverse clinical isolates. Here, for better understanding of the roles and characteristics of eDNA, we extracted ECM from *S*. *aureus* isolates grown in their respective optimal medium for biofilm formation (Fig. 1) and analysed eDNA by agarose gel electrophoresis (AGE) according to a recently published method^17^. Of note, our ECM extraction procedure did not cause cell lysis during experiments^17^.

**Figure 5:**
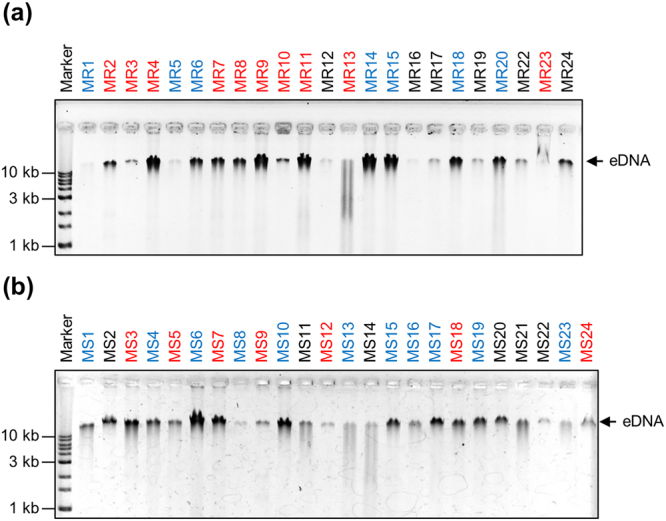
eDNA profiles in the ECM of *S*. *aureus* strains. eDNA was analysed by agarose gel electrophoresis (AGE) in extracted ECM from the indicated (**a**) MRSA and (**b**) MSSA strains cultured in the optimal medium for each strain. Molecular markers are shown at the extreme left of the gels. Strain names written in black, blue, and red represent low, medium, and high biofilm producers, respectively. All strains were cultured in the optimal medium required for biofilm formation as described in Fig. 1.

**Figure 6:**
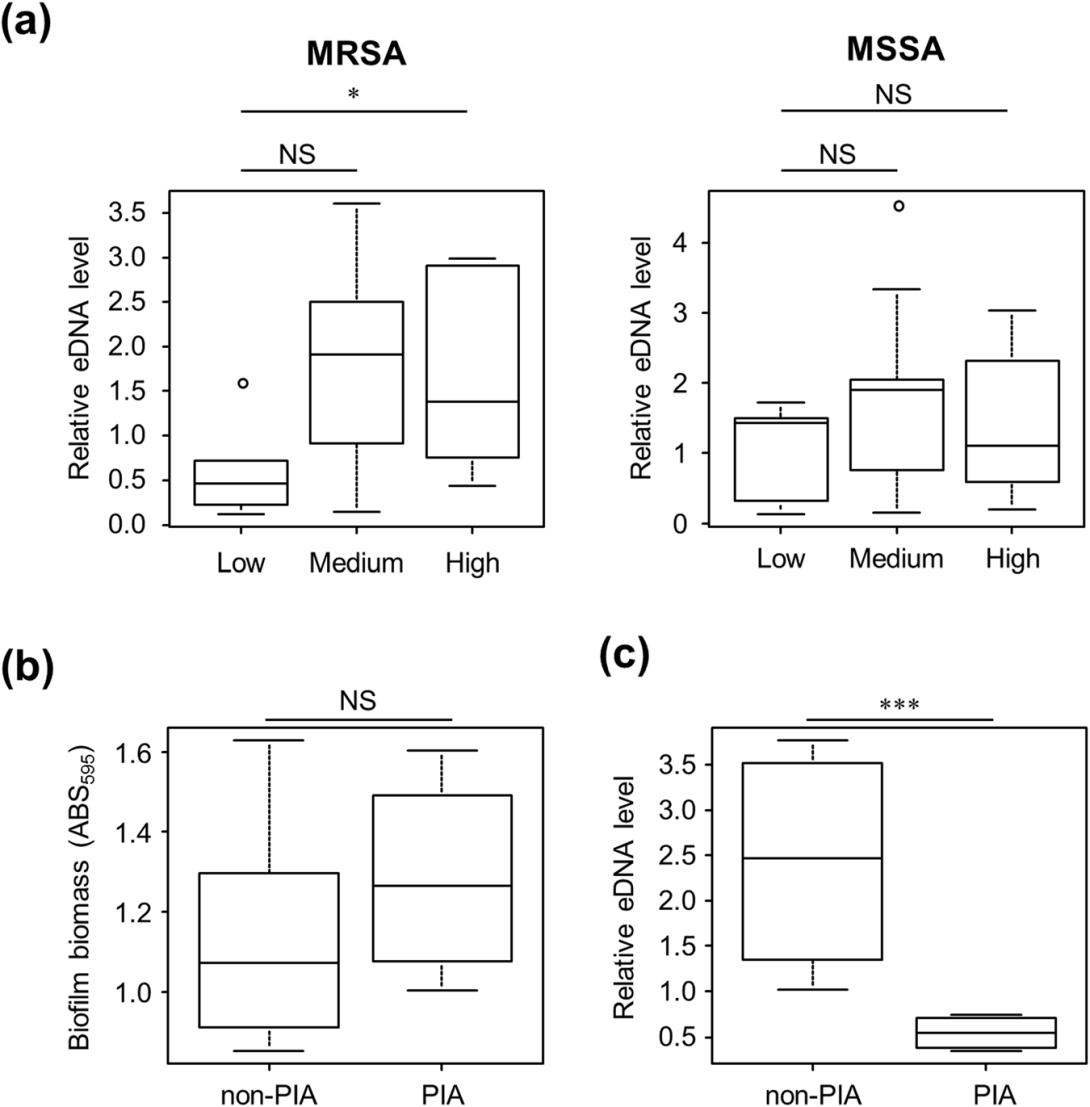
Relationship between Methicillin-resistance, eDNA level, biofilm-forming capacity, and PIA-dependency of biofilm formation. eDNA in the extracted ECM of the MRSA and MSSA strains were quantified. Band intensities on agarose gels (Fig. 5) were estimated. (**a**) Relationship between biofilm-forming capacity and eDNA level was analysed in MRSA and MSSA. The strains were categorised into three groups: low, medium, and high biofilm producers. (**b**) Relationship between biofilm-forming capacity and biofilm PIA-dependency was analysed in the biofilm high producers. The strains were categorised into two groups: PIA-independent (non-PIA) and -dependent (PIA) biofilm producers. (**c**) Relationship between eDNA level and PIA-dependency of biofilm formation was analysed in the biofilm high producers. The line in each box plot represents the median eDNA level of the indicated groups. ○, outlying values; *P < 0.05; ***P < 0.001; NS, not significant.

As shown in Fig. 5, eDNA was detected in all isolates of *S*. *aureus*. The band intensities of eDNA varied among the strains and some of them (MR13, MS13 and MS14) were partially degraded (Fig. 5). This degradation was presumably mediated by bacterial extracellular nucleases that have been shown to exert a negative impact on staphylococcal biofilm formation^18–20^. eDNA, isolated from MR23 strain, migrated more slowly on native agarose gels than did the eDNA from other strains (Fig. 5a), suggesting that in MR23, eDNA formed complexes with proteins or other macromolecules in the ECM. Interestingly, high biofilm producers of MRSA revealed higher eDNA levels as compared to low biofilm producers (Fig. 5a). For better understanding, we quantified band intensities of eDNA on agarose gel and performed statistical analysis. We found that eDNA was abundant in high biofilm producers of MRSA (Fig. 6a). However, there was no significant correlation between eDNA levels and biofilm formation capacity of MSSA (Fig. 6a). Similar results were obtained using an alternative method to quantify eDNA using a fluorescent probe (Fig. S4). These results imply that eDNA is generally required for generation of massive biofilms in MRSA. Additionally, certain low biofilm producers (MR24, MS2, MS20 and MS21) exhibited high quantities of eDNA, suggesting that the amount of eDNA does not always correlate with biofilm-forming capacity. Other factors (*e*.*g*., cell wall-anchored proteins, wall teichoic acids, phospholipids, other cellular components, and environmental factors, such as pH and metabolites) may also affect biofilm formation.

### Role of eDNA in PIA-dependent and -independent biofilms

It has been previously reported that a PIA-dependent MSSA biofilm showed resistance to DNase I treatment^13^. In contrast, other PIA-dependent MSSA and MRSA biofilms were inhibited or dispersed by DNase I treatment^14^. Our results demonstrated that all PIA-dependent massive MRSA and MSSA biofilms formed in the optimal medium were sensitive to DNase I treatment, but its effectiveness varied among strains (Figs 2 and 3). To understand the association of eDNA with PIA-dependency in biofilms of our collected strains, we compared eDNA levels between PIA-dependent and -independent biofilms. Although there was no significant difference between biofilm biomass and PIA-dependency (Fig. 6b), PIA-dependent biofilm producers revealed significantly lower eDNA levels than PIA-independent strains (Fig. 6c). Nevertheless, DNase I was active against PIA-dependent biofilms (Figs 2 and 3). These results indicate that PIA-dependent biofilms exhibit less but sufficient levels of eDNA to promote biofilm formation or to maintain biofilm structures.

It should be noted that the MR10-derived biofilm exhibited an extreme resistance to DNase I among the PIA-dependent biofilms (Figs 2 and 3). eDNA profiling revealed that the MR10 biofilm contained more or similar levels of eDNA as other PIA-dependent biofilm producers; MR3, MS9 and MS12 (Fig. 5). These results suggested that MR10 produces a large amount of PIA as compared to the other PIA-dependent strains, which may mask eDNA and protect it from the action of DNase I. To address this, we performed lectin-blot analysis using horseradish peroxidase-conjugated wheat germ agglutinin, which is commonly used to detect PIA in high biofilm producers of staphylococcal strains^21,22^. As shown in Fig. S5, MR10 produced a large amount of PIA and the level was drastically higher than that of the other strains.

These results highlight that contribution of eDNA in biofilm formation and structural integrity can depend on amount and patterns of other biofilm matrix components (*e*.*g*., PIA).

### Lack of correlation between eDNA level and biofilm-forming capacity

**Figure 7:**
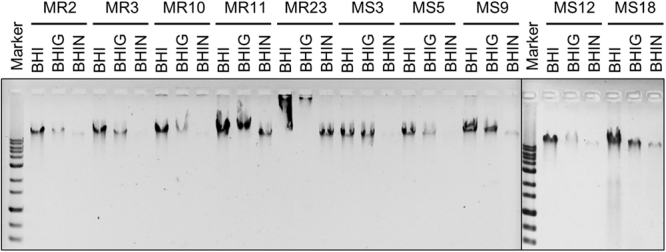
Effects of glucose and NaCl on production of eDNA. The indicated strains of MRSA and MSSA were cultured in BHI, BHIG, and BHIN media, and levels of eDNA in the extracted ECM were analysed by AGE as described in Fig. 5.

Our data showed that eDNA levels do not always correlated with biofilm-forming capacity of *S*. *aureus* strains (Fig. 6a). However, these comparisons were conducted using strains with different genetic backgrounds. To understand the correlation between eDNA level and biofilm-forming capacity of strains with same genetic backgrounds, we examined ECM components of the selected isolates (5 MRSA and 5 MSSA) grown in the different media (BHI, BHIG, and BHIN). Surprisingly, eDNA levels were generally enriched in the ECM of all tested strains when bacteria were cultured in BHI medium, whereas they were remarkably decreased in BHIG or BHIN media (Fig. 7). In contrast, biofilm production in these strains, other than MR10, was lower in BHI medium than in BHIG and BHIN media (Fig. 1). These data confirmed that the abundance of eDNA does not always correlate with the quantity of biofilm production. In addition, proteins present in ECM also exhibited similar trends, where a large amount and variety of proteins accumulated in the ECM when bacteria were cultured in BHI medium (Fig. S6). The quality, timing, and localisation (rather than abundance) of eDNA and other ECM components may also be important for biofilm formation.

## Discussion

In this study, we analysed biofilm-forming capacities of clinically isolated strains of *S*. *aureus*, a major causative agent of biofilm-related infections. The biofilm-forming capacity varied among strains, and the media composition drastically altered biofilm biomasses and major biofilm matrix components. Among the tested ECM-degrading enzymes, diverse biofilms showed the highest susceptibility to DNase I. ECM profiling demonstrated that eDNA was commonly present in the ECM and its level was distinct among strains and different culture conditions. These results indicated that eDNA is the most common ECM component in massive biofilms of *S*. *aureus*, although the abundance of eDNA is not always correlated with the biofilm-forming capacity. Therefore, eDNA could be used a primary target to eradicate *S*. *aureus* biofilms.

After glucose supplementation, approximately 89.4% of isolates were triggered to produce biofilms (Fig. 1). This was consistent with the previous report by O’Gara’s group^11^, in which 74% of 114 MRSA and 84% of 98 MSSA isolates were capable of biofilm development in BHIG medium. The presence of glucose is known to repress the *agr* quorum sensing system by decreasing the pH^23^, which leads to repression of extracellular protease production^12^. Low pH was also reported to trigger association of biofilm matrix proteins onto cell surfaces^24,25^ and to induce functional amyloid assembly^26^. Some of these mechanisms are applicable to explain the effects of glucose on biofilm production as observed in our study. In addition, supplementation of glucose altered the patterns of proteins in the ECM (Fig. S6), suggesting that glucose might promote or suppress the expression of certain specific proteins present in the ECM. Glucose also reduced eDNA levels in various biofilms (Fig. 7). Given that fragmentation of eDNA was not observed when the bacteria were grown in BHIG medium (Fig. 7), glucose might attenuate the release of eDNA from cells, rather than promote the production or activity of extracellular nuclease production.

NaCl also enhanced staphylococcal biofilm formation (Fig. 1). The NaCl-triggered biofilm formation was considerably more prevalent among MSSA than MRSA strains (Fig. 1). This was also consistent with the results of O’Gara’s group^11^. However, the mechanism(s) underlying the association between methicillin resistance and biofilm phenotypes remained unclear^27^. Addition of NaCl to BHI medium remarkably diminished proteins and eDNA in ECM (Figs 7 and S6). It is highly likely that 3% NaCl (a concentration of 513.4 mM) inhibits the association of proteins and eDNA onto the bacterial surface, which has been previously reported for NaCl concentrations ≥500 mM^17^. The statistical analysis revealed that PIA-dependent biofilm producers exhibited lower eDNA levels as compare to PIA-independent biofilm producers (Fig. 6c). In this analysis, PIA-independent strains were grown in BHIG medium, while 3 of 4 PIA-dependent strains (MR3, MS9, and MS12, except for MR10) were cultured in BHIN medium (Fig. 5). Given that eDNA levels in BHIN medium were lower than in BHIG medium (Fig. 7), the significant difference in eDNA levels between PIA-dependent and -independent strains was likely due to the distinct culture conditions.

Interestingly, only MR10 strain formed robust biofilms in BHI as well as TSB media without supplementation of glucose or NaCl. On the other hand, biofilm formed by MR10 strain was partially inhibited by NaCl. It was reported that NaCl promoted PIA production in *S*. *aureus*^11^, but the PIA production in MR10 was not affected by NaCl (Fig. S5). Meanwhile, eDNA and proteins in the ECM were decreased when MR10 was grown in BHIN medium, suggesting that in the presence of NaCl, the reduction of biofilm formation by MR10 was caused either by the decreases in eDNA and proteins in the ECM (Figs 7 and S6) or by other unknown mechanisms. In this strain, regulatory mechanisms of biofilm formation may have interesting mechanistic implications that are needed to be clarified in the future.

ECM-profiling data indicated that ECM components, such as proteins and eDNA were more enriched when *S*. *aureus* strains were grown in BHI than in BHIG medium (Figs 7 and S6). These intracellular components might be released from bacterial cells via PSM-peptides induced cell leakage^28^, production of membrane vesicles^29^, or other mechanism(s). Among them, production of PSM-peptides is positively regulated by the *agr* system^28^. Therefore, it is reasonable to assume that supplementation of glucose induces acidification of media, which can inactivate the *agr* system^24^, leading to the reduction of the PSM peptides-derived excretion of cytoplasmic substances. However, the biofilm biomass was less when they were cultured in BHI than in BHIG medium (Fig. 2). It has been reported that when *S*. *aureus* strains were cultured in BHI medium with glucose, metabolism of glucose led to the accumulation of acetic acid and the final pH of the medium was less than 5^30^. In contrast, without glucose supplementation, the pH level of BHI culture initially reduced to 6.5, whereas the final pH of the culture was higher than 8.5^30^. At alkaline pH, biofilms may be unstable^31^ and the biofilm biomass could reduce drastically in BHI medium. Therefore, in addition to ECM components, acidification of the growth medium is also important for determination of biofilm biomass.

Structural integrity of biofilms is maintained by various molecules. Therefore, it is reasonable to consider that many biofilms show sensitivity to not only one enzyme, but also to others. Indeed, various biofilms were inhibited and/or dispersed by several enzymes (Figs 2 and 3). In contrast, certain biofilms exhibited specific sensitivity to one enzyme, but resistance to the others. For example, biofilm formed by MR10 strain was significantly inhibited or dispersed by Dispersin B, whereas Proteinase K and DNase I were less effective (Figs 2 and 3). These results indicated that PIA is a major contributor to the biofilm formed by MR10. In addition, Proteinase K was a potent antagonist of MR23-derived biofilm, whereas this film was mildly inhibited by DNase I, suggesting that proteins are dominant components in MR23-derived biofilm. Interestingly, ECM profiles supported this notion. MR10 contained a large quantity of PIA in the ECM (Fig. S5), while MR23 displayed a unique and abundant protein, namely extracellular adherence protein (Eap) (Fig. S6). In a previous study, we demonstrated that Eap promoted biofilm formation by *S*. *aureus*^15^. When a large amount of PIA or Eap is present, a single agent alone might establish a robust biofilm by compensating for roles of other components, such as eDNA, or by preventing the action of certain ECM-degrading enzymes. In addition, redundancy that exists in diverse biofilm formation mechanisms occasionally obscures details of the pathways involved in such processes^32^. In future studies, comprehensive approaches, such as transcriptomics and proteomics, will be required to identify the key components, and to understand the mechanisms underlying the development and structural integrity of biofilms. These investigations would lead to the development of effective anti-biofilm therapies and technologies that would target specific or multiple ECM components using different enzymes or small inhibitor compounds.

## Methods

### Bacterial strains and media

Bacterial strains employed in this study, were isolated from patients in The Jikei University Hospital (Table S1). The frozen stock of MR21 strain was found to be contaminated during isolation or preservation processes. Therefore, the isolate was omitted in this study. Bacteria were grown in BHI (Becton Dickinson, Franklin Lakes, NJ, USA) and TSB media (Becton Dickinson). If required, media were supplemented with glucose or NaCl at the indicated concentrations.

### Enzymes

Dispersin B was purchased from Kane Biotech Inc. (Manitoba, Canada), DNase I (protease-free) was obtained from Roche Diagnostics (Mannheim, Germany), and Proteinase K was procured from Sigma-Aldrich (St. Louis, MO, USA).

### Biofilm formation

Overnight cultures grown in BHI medium at 37 °C were diluted 1,000-fold in BHI, BHI supplemented with 1% (w/v) glucose (BHIG), BHI supplemented with 3% (w/v) NaCl (BHIN), TSB, TSB supplemented with 1% (w/v) glucose (TSBG), or TSB supplemented with 4% (w/v) NaCl (TSBN). Two hundred microlitres of these suspensions were cultured in 96-well flat bottom polystyrene plates (Corning, NY, USA) at 37 °C for 24 h. These biofilms were then washed twice with 150 μl of phosphate-buffered saline (PBS) and stained with 0.05% (w/v) Crystal Violet (CV) (150 μl) for about 3 min at 25 °C. After staining, biofilms were washed once with PBS and biofilm biomass was quantified by measuring the absorbance at 595 nm with an Infinite F200 Pro (Tecan, Männedorf, Switzerland) microtitre plate reader.

### Isolation and characterisation of ECM

ECM was extracted from bacteria grown under biofilm formation conditions as described previously^17^. Unless otherwise noted, the optimal medium required for biofilm formation by each strain (Fig. 1) was used for bacterial culture and extraction of ECM. Proteins and PIA in ECM were analysed by SDS-PAGE followed by Coomassie brilliant blue (CBB) staining. eDNA was analysed by AGE and visualised by staining with ethidium bromide and gel images were recorded with a LAS-4000 luminescent image analyser (GE Healthcare, Buckinghamshire, UK). Amounts of eDNA were quantified using the ImageQuant image analyser software (GE Healthcare). Quantities of the extracted ECM were normalized to wet weights of the harvested biofilms.

### Enzyme susceptibility of biofilms

To perform biofilm inhibition assays, Proteinase K (100 μg/ml), DNase I (100 U/ml), or Dispersin B (20 μg/ml) were added to culture media at the onset of biofilm formation. For biofilm destruction assays, enzymes at the same concentrations were added directly to 24 h-old biofilms without removal of the culture medium and the mixtures were incubated for 2 h at 37 °C. The biofilms were quantified as described above.

### Statistical analysis

Student’s *t* test was used to assess biofilm formation and enzyme susceptibility using Microsoft Excel software. Chi-square analysis was performed to determine significant differences in NaCl-promoted biofilm formation between MRSA and MSSA strains using Microsoft Excel. Statistical analysis of biofilm formation was also performed using EZR software^33^. For multiple group comparisons (comparison of biofilm formation in bacterial subtypes, assessment of eDNA levels in the ECM and biofilm-forming capacity, and investigation of PIA-dependency and eDNA levels), Kruskal-Wallis one-way analysis of variance with Dunn’s multiple comparison post-hoc analysis, or Mann-Whitney U tests were used to determine whether any of the groups exhibited statistically significant different capacities to form biofilms. Pearson correlation coefficients were determined among anti-biofilm activities of the indicated enzymes. For all analyses, a *P*-value of <0.05 was considered statistically significant.

## Electronic supplementary material

Supplementary Information
