# Supplementary material for: Broad impact of extracellular DNA on biofilm formation by clinically isolated Methicillin-resistant and -sensitive strains of Staphylococcus aureus
Source: Sci Rep. 2018 Feb 2;8:2254. doi: 10.1038/s41598-018-20485-z (PMC5797107; doi:10.1038/s41598-018-20485-z)

Supplementary Information

Broad impact of extracellular DNA on biofilm formation by clinically isolated Methicillin-resistant and -sensitive strains of *Staphylococcus aureus*

Shinya Sugimoto[1,2†*], Fumiya Sato[1,3†], Reina Miyakawa[1], Akio Chiba[1,2], Shoichi Onodera[3], Seiji Hori[3], and Yoshimitsu Mizunoe[1,2]

[1] Department of Bacteriology, The Jikei University School of Medicine 3-25-8, Nishi-Shimbashi, Minato-ku, Tokyo, Japan.
[2] Jikei Center for Biofilm Research and Technology, The Jikei University School of Medicine 3-25-8, Nishi-Shimbashi, Minato-ku, Tokyo, Japan.
[3] Department of Infectious Disease and Control, The Jikei University School of Medicine 3-25-8, Nishi-Shimbashi, Minato-ku, Tokyo, Japan.

[†] Both authors contributed equally to the study.
[*] For correspondence. E-mail: ssugimoto@jikei.ac.jp; Tel: (+81) 3 3433 1111; Fax: (+81) 3 3433 1111.

Running title: Importance of eDNA in diverse *Staphylococcus aureus* biofilms

SUPPLEMENTARY METHODS

Construction of strain

Mutant strain of *S. aureus* RN4220 were constructed using the *E. coli*-*S. aureus* shuttle vector pKOR1[1] according to the procedure modified by Chiba *et al.*[2]. In brief, approximately 500-bp upstream and downstream sequences of the *icaA* gene was amplified by PCR from the RN4220 genomic DNA using the following primer sets: *attB1-icaA*-F (5'-GGGGACAAGTTTGTACAAAAAAGCAGGCTGAATTGATATAAAGCATCAATTGAATAG-3') and *icaA*-R (5'-CTGTTTCTCTTACAATATTTAGCGATGATTTTTCTTTACCTACCTTTCGTTAGTTAGGTT-3'); *icaA*-F (5'-AACCTAACTAACGAAAGGTAGGTAAAGAAAAATCATCGCTAAATATTGTAAGAGAAACAG-3') and *attB2-icaA*-R (5'-GGGGACCACTTTGTACAAGAAAGCTGGGTCAGAAAATTCGCTTTTCTTACAC-3'). These fragments were connected by splicing by overlap extension PCR (SOE-PCR)[3]. The created PCR product was cloned into pKOR1 using the Gateway BP Clonase II enzyme mix (Life Technologies, Palo Alto, CA, USA); the resulting plasmids are referred to as pKOR1-Δ*icaA*. Using this plasmid, the *icaA* gene was deleted solely or sequentially from the RN4220 genomic DNA according to the procedure reported previously[1,2].

For deletion of *icaA* from MR10, following primer sets were used: icaA_F (5'-GGGGACAAGTTTGTACAAAAAAGCAGGCTGAATTGATATAAAGCATCAATTGAATAG-3') and SOE-R (5'-TCTCTTACAATATTTAGCGATGATTATTTATGTCAGGCTTCTTGTTCAAT-3') and icaA_R (5'-GGGGACCACTTTGTACAAGAAAGCTGGGTC AGAAAATTCGCTTTTCTTACAC-3') and SOE-F (5'-ATTGAACAAGAAGCCTGACATAAATAATCA TCGCTAAATATTGTAAGAGA-3'). Other procedures were similar to the construction of RN4220 Δ*icaA*.

Characterisation of the genetic background

The *agr* types were determined by a multiplex PCR assay as described previously[4]. The multi-locus sequence typing (MLST) types were determined to detect the following seven housekeeping genes: carbamate kinase (*arcC*), shikimate dehydrogenase (*aroE*), glycerol kinase (*glpF*), guanylate kinase (*gmk*), phosphate acetyltransferase (*pta*), triosephosphate isomerase (*tpi*), and acetyl coenzyme A acetyltransferase (*yqiL*)[5]. The

sequences of the PCR products were compared with the existing sequences available from the website (http://saureus.mlst.net/). The MLST types were determined at the allelic query site (http://saureus.mlst.net/sql/allelicprofile_choice.asp).

Hemolytic activity assay

An overnight culture (5 μL) of the indicated strains was spotted onto a sheep blood agar plate (Eiken Kagaku, Tokyo, Japan). The plate was incubated at 37°C overnight and stored at 4°C for additional 24 h. Pictures were taken using digital camera and hemolytic activities were judged by formation of halo around colonies.

Quantification of eDNA in the extracellular matrix (ECM)

Concentration of eDNA in the isolated ECM was measured using Qubit dsDNA BR Kit (Thermo Fisher Scientific Inc., Waltham, MA, USA) according to the manufacture's instruction.

Lectin blotting

To detect PIA in isolated ECM, lectin blotting was conducted using HRP-WGA (Vector Laboratories, Burlingame, CA, USA). The isolated ECM was electrophoretically separated by SDS-PAGE and transferred to a PVDF membrane. The membrane was treated with a blocking solution containing 3% BSA in TSB-T for 1 h at 25°C. After gentle washing with TBS-T, the membrane was probed with HRP-WGA (5 ng/ml in TBS-T) for 1 h at 25°C. After washing three times with TBS-T, PIA-related signals were detected using ECL Prime on LAS-4000.

SUPPLEMENTARY FIGURE LEGENDS

Figure S1. Biomass of biofilms formed by control *S. aureus* strains under different culture conditions. Biofilms of *S. aureus* RN4220 wild-type and its isogenic Δ*icaA* strains were formed in the indicated media at 37°C for 24 h. BHIG: brain heart infusion (BHI) supplemented with glucose, BHIN: BHI supplemented with NaCl, TSBG: tryptic soy broth (TSB) supplemented with glucose, and TSBN: TSB supplemented with NaCl. Y-axis represents biofilm biomass determined by measuring absorbance of CV-stained

biofilms at 595 nm. Data are represented as mean ± standard deviation from at least three independent experiments.

Figure S2. Comparison of biofilm-forming capacity, antibiotic resistance and staphylococcal species. Comparison of biofilm-producing capacity between MRSA and MSSA grown in media supplemented with glucose or NaCl for biofilm formation in each strain. The line in each box plot represents the median biofilm-forming capacity (biofilm biomass determined in Fig. 1) of the indicated groups. NS, not significant.

Figure S3. Analysis for correlation between biofilm-forming capacity and the *agr* types. Statistical analyses were performed to compare biofilm-forming capacity among the *agr* types in *S. aureus*. The line in each box plot represents the median biofilm-forming capacity (biofilm biomass determined in Fig. 1) of the indicated groups. ○, Outlying values. There was no statistically significant difference in all multiple comparison analyses.

Figure S4. Quantification of eDNA in the isolated ECM. eDNA in the ECM of the MRSA and MSSA strains were quantified using Qubit dsDNA BR Kit. The strains were categorized into three groups: low, medium and high biofilm producers. The line in each box plot represents the median eDNA level of the indicated groups. ○, Outlying values. *, P <0.05. NS, not significant.

Figure S5. Identification of PIA in the isolated ECM. ECM isolated from the indicated strains with high biofilm-forming capacity were separated by SDS-PAGE and transferred to a membrane. The membrane was probed with HRP-WGA to detect PIA staked on the top of the gel. MR10 wild-type and its isogenic Δ*icaA* strains grown in BHI, BHIG, or BHIN were used as a positive and a negative control, respectively. Sensitivities of the biofilms to the ECM-degrading enzymes are also indicated. Upper cases indicate the culture media used, and otherwise, the strains were grown in BHIG medium. S, sensitive.

Figure S6. Effects of glucose and NaCl on profiles of proteins in the ECM. The indicated strains of MRSA (**a**) and MSSA (**b**) were cultured in BHI, BHIG and BHIN, and proteins in the ECM were analysed by SDS-PAGE with CBB staining.

SUPPLEMENTARY REFERENCES

1. Bae, T. & Schneewind, O. Allelic replacement in *Staphylococcus aureus* with inducible counter-selection. *Plasmid* **55**, 58-63 (2006).
2. Chiba, A., Sugimoto, S., Sato, F., Hori, S. & Mizunoe, Y. A refined technique for extraction of extracellular matrices from bacterial biofilms and its applicability. *Microb. Biotechnol.* **8**, 392-403 (2015).
3. Ho, S.N., Hunt, H.D., Horton, R.M., Pullen, J.K. & Pease, L.R. Site-directed mutagenesis by overlap extension using the polymerase chain reaction. *Gene* **77**, 51-59 (1989).
4. Bibalan, M.H., Shakeri, F., Javid, N., Ghaemi, A. & Ghaemi, E.A. Accessory gene regulator types of *Staphylococcus aureus* isolated in Gorgan, North of Iran. *J. Clin. Diagn. Res.* **8**, DC07-9 (2014).
5. Enright, M.C., Day, N.P., Davies, C.E., Peacock, S.J. & Spratt, B.G. Multilocus sequence typing for characterization of methicillin-resistant and methicillin-susceptible clones of *Staphylococcus aureus*. *J. Clin. Microbiol.* **38**, 1008-1015 (2000).
6. Kreiswirth *et al*. The toxic shock syndrome exotoxin structural gene is not detectably transmitted by a prophage. *Nature* **305**, 709-712 (1983).
7. Sugimoto, S. *et al*. *Staphylococcus epidermidis* Esp degrades specific proteins associated with *Staphylococcus aureus* biofilm formation and host-pathogen interaction. *J. Bacteriol.* **195**, 1645-1655 (2013).
8. Sugimoto, S., Iwase, T., Sato, F., Tajima, A., Shinji, H. & Mizunoe, Y. Cloning, expression and purification of extracellular serine protease Esp, a biofilm-degrading enzyme, from *Staphylococcus epidermidis*. *J. Appl. Microbiol.* **111**, 1406-1415 (2011).

Table S1. Bacterial strains used in this study

S. aureus strains	Isolation sites and description	References/sources
Laboratory strain		
RN4220	Restriction deficient derivative of 8325-4	6
RN4220*icaA*	Δ*icaA* strain	This study
Clinical strains[a]		
MRSA		
MR1	Wound	This study
MR2	Ear	This study
MR3	Wound	This study
MR4	Nasal cavity	This study
MR5	Sputum	This study
MR6	Throat	This study
MR7	Throat	This study
MR8	Throat	This study
MR9	Sputum	This study
MR10	Urinary catheter	2, 7
MR11	Skin	This study
MR12	Sputum	This study
MR13	Throat	This study
MR14	Pus	This study
MR15	Throat	This study
MR16	Sputum	This study
MR17	Wound	This study
MR18	Sputum	This study
MR19	Sputum	This study
MR20	Sputum	This study
MR22	Urine	This study
MR23	Pus	2, 7, 8
MR24	Urine	This study
MR10 Δ*icaA*	Δ*icaA* strain	This study
MSSA		
MS1	Throat	This study
MS2	Wound	This study
MS3	Urinary catheter	This study
MS4	Sputum	7
MS5	Throat	This study
MS6	Vagina	This study
MS7	Ear	This study
MS8	Vagina	This study
MS9	Urine	This study
MS10	Blood	2, 7
MS11	Wound	This study
MS12	Urine	This study
MS13	Wound	This study
MS14	Ear	This study
MS15	Feces	This study
MS16	Blood	This study
MS17	Pus	This study
MS18	Pus	This study
MS19	Sputum	This study
MS20	Sputum	This study
MS21	Ear	This study
MS22	Sputum	This study
MS23	Pus	This study
MS24	Paranasal sinus	This study

[a]All strains were isolated from patients in The Jikei University Hospital.

Table S2. Sequence types, hemolytic activities and biofilm phenotypes of *S. aureus* clinical strains used in this study

Bacterial strains	MLST types	*agr* types	hemolytic activity[a]	Biofilm-forming capacity with glucose[b]	Biofilm-forming capacity with NaCl[b]	Biofilm PIA-dependency[c]
**MRSA**						
MR1	764	II	+	M	L	n.d.
MR2	89	III	+ +	H	L	−
MR3	5	II	−	M	H	+
MR4	1	III	+ +	H	L	−
MR5	5	III	−	M	M	n.d.
MR6	5	III	−	M	M	n.d.
MR7	5	III	−	H	L	−
MR8	5	III	−	H	L	−
MR9	5	II	+ +	H	L	−
MR10	5	II	+	H	M	+
MR11	91	III	+ +	H	L	−
MR12	5	II	−	L	L	n.d.
MR13	5	II	−	H	L	−
MR14	149	II	+ +	M	L	n.d.
MR15	149	II	+ +	M	L	n.d.
MR16	764	II	+	L	L	n.n.
MR17	64	III	+	L	L	n.d.
MR18	5	III	−	M	L	n.d.
MR19	764	II	+	L	L	n.d.
MR20	2613	II	−	M	L	n.d.
MR22	64	II	+	L	L	n.d.
MR23	380	I	+ +	H	L	−
MR24	5	II	+	L	L	n.d.
**MSSA**						
MS1	25	I	−	M	L	n.d.
MS2	432	I	+	L	L	n.d.
MS3	20	I	−	H	L	+
MS4	8	I	−	M	L	n.d.
MS5	6	I	−	H	L	+
MS6	15	II	+ +	M	L	n.d.
MS7	5	II	+ +	H	M	+
MS8	72	I	+ +	L	M	n.d.
MS9	5	II	+	M	H	+
MS10	30	III	−	M	L	n.d.
MS11	630	I	+ + +	L	L	n.d.
MS12	45	I	+	M	H	+
MS13	15	II	+ + +	M	L	n.d.
MS14	25	I	+ +	L	L	n.d.
MS15	45	I	−	M	L	n.d.
MS16	12	II	−	M	L	n.d.
MS17	623	I	−	M	L	n.d.
MS18	188	I	+ +	H	L	−
MS19	188	I	−	M	L	n.d.
MS20	45	I	−	L	L	n.d.
MS21	12	II	+	L	L	n.d.
MS22	121	IV	+ + +	L	L	n.d.
MS23	15	II	+ +	M	L	n.d.
MS24	30	III	−	H	L	+

[a]+, unclear halo. ++, clear but small halo. +++, clear and large halo. −, no halo

[b]Determined in Fig. 1. H, high; M, medium; L, low.

[c]The Dispersin B-susceptible biofilm was considered to be a PIA-dependent biofilm as determined in Figs. 2 and 3. +, PIA-dependent biofilm. −, PIA-independent biofilm. n.d., not determined.

Sugimoto *et al*. Figure S1

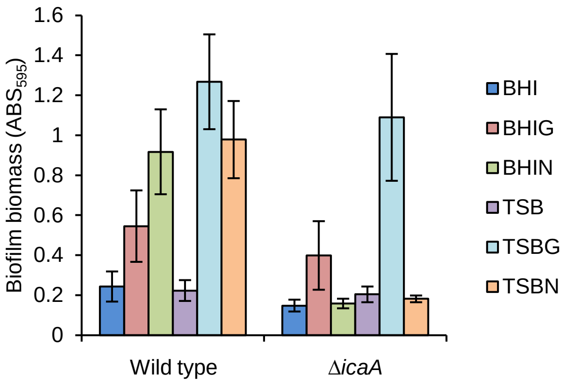

Sugimoto *et al*. Figure S2

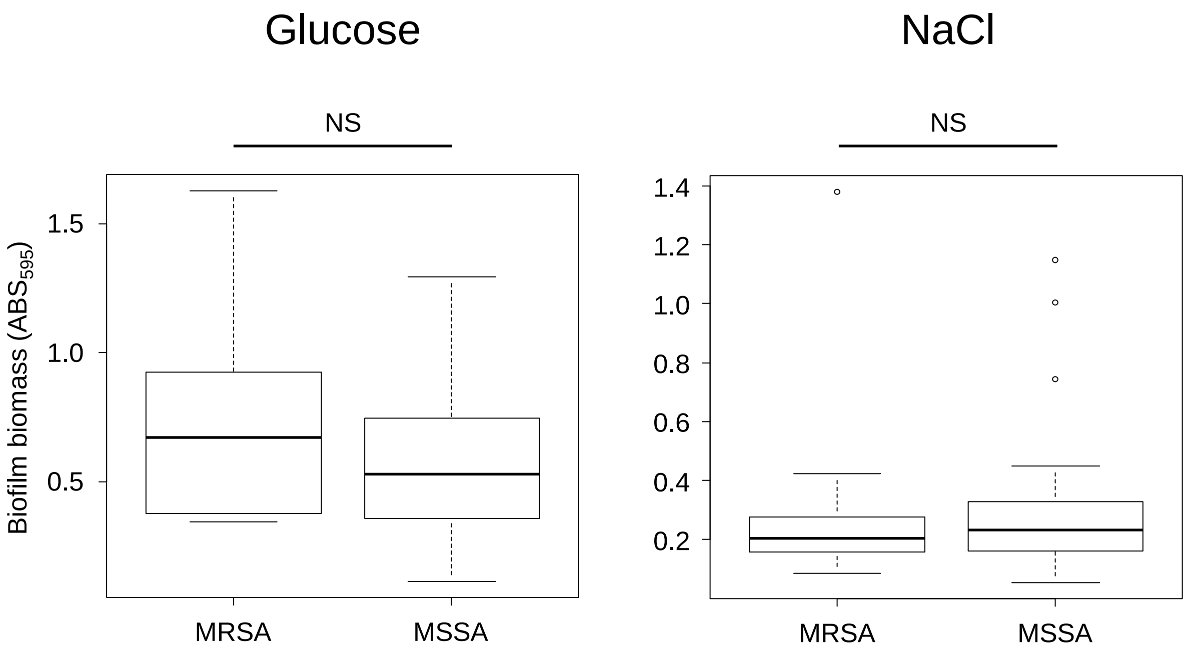

Sugimoto *et al*. Figure S3

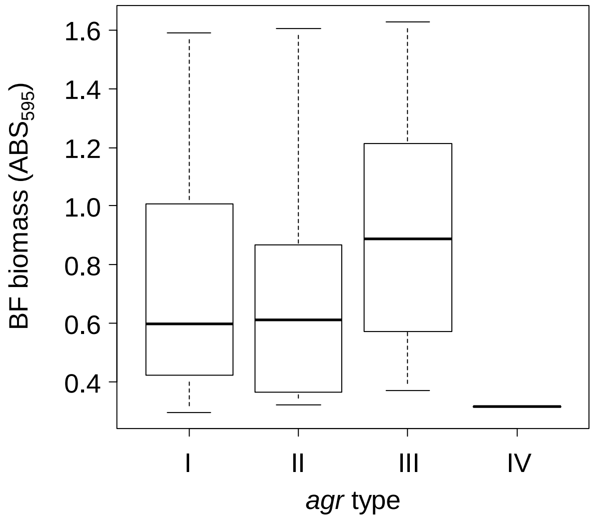

Sugimoto *et al*. Figure S4

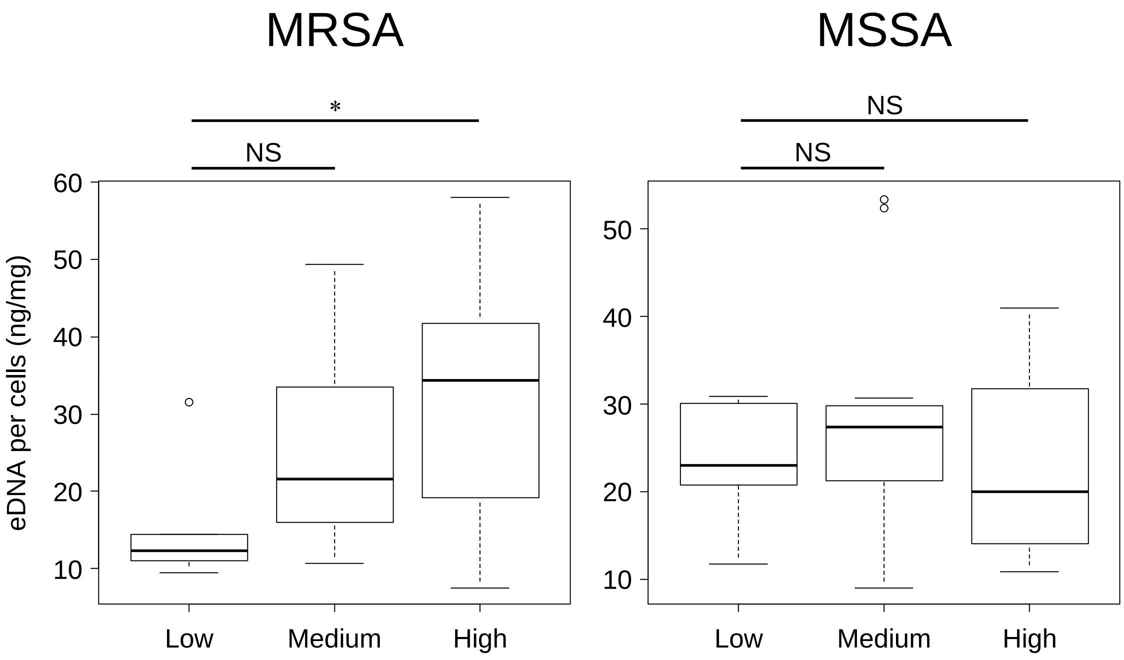

Sugimoto *et al*. Figure S5

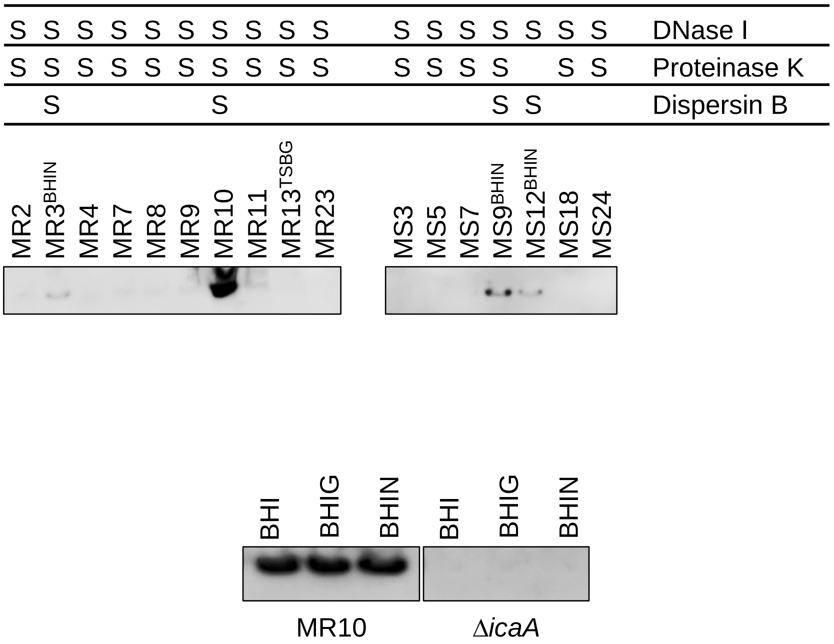

Sugimoto *et al*. Figure S6

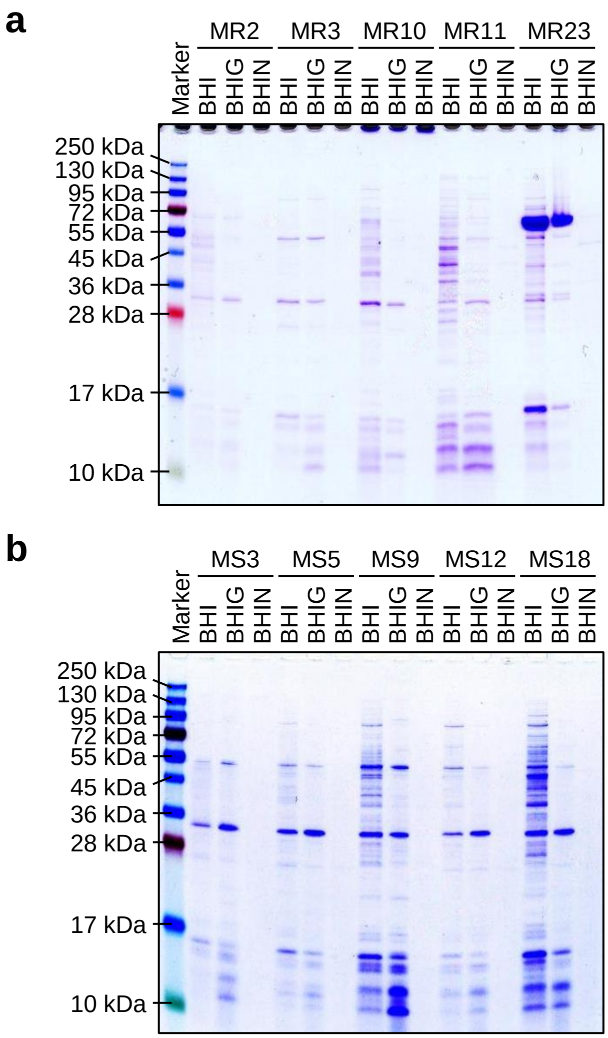

Supplement: Supplementary file 1 — Supplementary Information [file 41598_2018_20485_MOESM1_ESM.pdf]
